# A randomized controlled trial in schools aimed at exploring mechanisms of change of a multifaceted implementation strategy for promoting mental health at the workplace

**DOI:** 10.1186/s13012-022-01230-7

**Published:** 2022-09-01

**Authors:** Lydia Kwak, Anna Toropova, Byron J. Powell, Rebecca Lengnick-Hall, Irene Jensen, Gunnar Bergström, Liselotte Schäfer Elinder, Kjerstin Stigmar, Charlotte Wåhlin, Christina Björklund

**Affiliations:** 1 Unit of Intervention and Implementation Research for Worker Health, Institute for Environmental Medicine, Karolinska Institutet, Stockholm, Sweden; 2 Center for Mental Health Services Research, Brown School, Washington University in St. Louis, St. Louis, Missouri USA; 3 Center for Dissemination & Implementation, Institute for Public Health, Washington University in St. Louis, St. Louis, Missouri USA; 4 Division of Infectious Diseases, John T. Milliken Department of Medicine, School of Medicine, Washington University in St. Louis, St. Louis, Missouri USA; 5 Department of Occupational and Public Health Sciences, Faculty of Health and Occupational Studies, Centre for Musculoskeletal Research, University of Gävle, Gävle, Sweden; 6 Department of Global Public Health, Karolinska Institutet, Stockholm, Sweden; 7 Centre for Epidemiology and Community Medicine, Stockholm, Stockholm Region Sweden; 8 Department of Health Sciences, Lund University, Lund, Sweden; 9 Occupational and Environmental Medicine Center, Department of Health, Medicine and Caring Sciences, Division of Prevention, Rehabilitation and Community Medicine, Linköping University, Linköping, Sweden

**Keywords:** Implementation mechanism, Guideline, Implementation strategies, Schools, Mediators, COM-B, Theoretical Domains Framework

## Abstract

**Background:**

This study will explore implementation mechanisms through which a single implementation strategy and a multifaceted implementation strategy operate to affect the implementation outcome, which is fidelity to the *Guideline For The Prevention of Mental Ill Health* within schools. The guideline gives recommendations on how workplaces can prevent mental ill health among their personnel by managing social and organizational risks factors in the work environment. Schools are chosen as the setting for the study due to the high prevalence of mental ill health among teachers and other personnel working in schools. The study builds on our previous research, in which we compared the effectiveness of the two strategies on fidelity to the guideline. Small improvements in guideline adherence were observed for the majority of the indicators in the multifaceted strategy group. This study will focus on exploring the underlying mechanisms of change through which the implementation strategies may operate to affect the implementation outcome.

**Methods:**

We will conduct a cluster-randomized-controlled trial among public schools (*n*=55 schools) in Sweden. Schools are randomized (1:1 ratio) to receive a multifaceted strategy (implementation teams, educational meeting, ongoing training, Plan-Do-Study-Act cycles) or a single strategy (implementation teams, educational meeting). The implementation outcome is fidelity to the guideline. Hypothesized mediators originate from the COM-B model. A mixed-method design will be employed, entailing a qualitative study of implementation process embedded within the cluster-randomized controlled trail examining implementation mechanisms. The methods will be used in a complementary manner to get a full understanding of the implementation mechanisms.

**Discussion:**

This implementation study will provide valuable knowledge on how implementation strategies work (or fail) to affect implementation outcomes. The knowledge gained will aid the selection of effective implementation strategies that fit specific determinants, which is a priority for the field. Despite recent initiatives to advance the understanding of implementation mechanisms, studies testing these mechanisms are still uncommon.

**Trial registration:**

ClinicalTrials.org dr.nr 2020-01214.

**Supplementary Information:**

The online version contains supplementary material available at 10.1186/s13012-022-01230-7.

Contributions to the literature

- This study contributes with knowledge on the processes and steps involved in specifying mechanisms of change.
- Findings will advance our knowledge on how and why implementation strategies work.
- This study will fill identified research gaps in specifying and examining implementation mechanisms in general and in the Swedish school context.

## Introduction

### The importance of creating a sustainable working environment in schools

A professional group that has a high prevalence of mental-ill health, and related presenteeism and sick leave, are teachers [1, 2]. Teachers’ work environment is characterized by high workload, role overload, increased class size per teacher, and lack of support from management, resulting in a high risk for mental ill health [1–3]. One way of preventing work-related mental ill health is to apply a systematic approach to the management of organizational and social risks within the work environment as recommended by the Swedish Agency for Work Environment and Health. Many schools in Sweden, however, lack such as systematic approach [4]. To support workplaces, including schools, with the management of their social and organizational work environment and the prevention of mental ill health, we launched the *Guideline For The Prevention of Mental Ill-Health At The Workplace* [5]. The guideline is based on the best available evidence (e.g., [6]) and has been compiled through a practice-based research network including employers, occupational health services staff, and researchers.

### Supporting the implementation of the guideline within schools

Even though guidelines are an essential part of achieving sustainable working environments, it is well known that solely disseminating guidelines rarely results in full implementation in practice [7, 8]. Between 2017 and 2019, we conducted a cluster-randomized controlled trial with the aim to support schools with the implementation of the guideline to prevent mental ill health. We developed a multifaceted implementation strategy containing an educational meeting, ongoing training through workshops, implementation teams, and Plan-Do-Study-Act cycles [9]. The effectiveness of the multifaceted strategy was compared with a discrete implementation strategy (educational meeting) among 19 schools in Sweden. Small improvements in guideline adherence were observed for the majority of the indicators in the multifaceted strategy group; however, improvements were not statistically significant from the discrete strategy group [10]. One of the reasons behind the lack of effectiveness could be the large organizational changes that occurred in some of the participating schools. This was confirmed by the sensitivity analysis in organizationally stable schools, which demonstrated larger and more consistent improvements. To further understand the (lack) of effectiveness of the multifaceted implementation strategy, we will conduct a new cluster-randomized controlled trial to explore the underlying mechanisms of change through which the implementation strategy may operate to affect the implementation outcome. The need for understanding how and why implementation strategies work as well as to which extent has been highlighted in several studies [11–13]. Despite recent initiatives to advance the understanding of implementation mechanisms [11], studies testing these mechanisms are still uncommon [12, 13]. An important prerequisite for exploring mechanism of change is selecting implementation strategies based on a systematic approach. This includes the identification of barriers and facilitators, and the selection of implementation strategies that address the identified barriers and facilitators. Several existing methods and frameworks can be leveraged to support researchers and planners in executing a more systematic approach [14–17].

### Steps for testing specific mechanisms of change

The current effort to specify mechanisms of change builds directly on our previous trial [9]. The first step was the specification of target-behaviors related to the recommendations of the guideline and the identification of barriers and facilitators [9]. First, the barriers were identified from a European survey conducted by the Organization for Economic Co-operation and Development (OECD) on barriers that hinder organizations from managing organizational and social risks [18]. The main barriers identified included the lack of knowledge and lack of guidance on how to manage organizational and social risk factors in the work environment [18]. To supplement these survey findings, planning workshops were conducted with school principals to identify barriers to implementing the *Guideline For The Prevention of Mental Ill-Health At The Workplace* within their school [9]. Barriers identified by the principals included the lack of knowledge on how to manage organizational and social risks at the workplace, unclear professional roles regarding who has the responsibility for the prevention of mental ill health within the school, lack of support from staff and school district, and difficulty prioritizing the prevention of mental ill health due to lack of time. An important facilitator identified by the principals was the need for a systematic approach (working with the work environment routinely) to implementing the guideline recommendations in their workplace [9].

In the second step, we selected the COM-B as a model to inform the pathways of change. The COM-B model postulates that for a behaviour to occur, a person must have the capability, opportunity, and motivation to perform the behaviour in question [19]. Capability refers to whether an individual has the necessary knowledge, skills, and ability to perform the behavior [19]. Opportunity relates to factors that are external to the individual that make the performance of the behavior possible and can be divided into physical opportunity, including time and resources, and social opportunity, such as social support and social norms [19]. Motivation refers to internal processes that influence decision making and behavior, including making plans [19]. Following the pathways of change proposed by the COM-B model, the identified barriers and facilitators were organized and structured according to the COM-B constructs and applied to the principals’ role. It was hypothesized that principals needed to have the capability to engage in the behaviour (i.e., have knowledge and skills related to the prevention of mental ill health in accordance with the guideline); the opportunity to engage in the behaviour (i.e., have the time to engage in the behavior, prioritize the behavior, receive support from staff and school district and have clearly defined roles; and have the motivation to perform the behavior (i.e., decision to implement the guideline through planning and structure).

In the third step, implementation strategies were selected to overcome the barriers and enable the facilitators. The selection was informed by existing compilations of implementation strategies (e.g., [20–22]. In consensus with experts and principals, implementation strategies were selected by matching strategies from the compilations with the determinants related to the three constructs of the COM-B model [9]. For example, the strategies of conducting educational meetings and ongoing training were chosen to address determinants related to capability (i.e., knowledge and skills). There is evidence that educational meetings and workshops can impact professional behavior [23] by providing access to knowledge and information [20]. Conducting ongoing training can also be used to provide individuals with skills to perform the behavior [24].

The formation of local implementation teams was a strategy chosen to address determinants related to opportunity and to provide support to the principal. Even though the evidence for the effectiveness of implementation teams is limited, implementation teams have been identified as a critical component for facilitating implementation by the Quality Implementation Framework [25]. Implementation teams create an internal support structure for implementation by specifying who will perform the tasks related to delivering the intervention and monitoring the implementation process [25]. A core function of implementation teams is to conduct improvement cycles, such as Plan-Do-Study-Act cycles (PDSA-cycles [26]. Implementation teams employ PDSA-cycles to identify, problem-solve, and address barriers and improve implementation [26]. PDSA-cycles can be used as a strategy to address opportunity (i.e., by creating an environmental context and resources for implementation), capability (i.e., increasing self-efficacy by conducting small changes), and motivation (i.e., by facilitating planning and decision-making) [20].

A strategy addressing opportunity was added to the multifaceted strategy based on findings of our process evaluation (unpublished observations). The process evaluation conducted parallel to our previous study [9] identified the lack of support from the school district as an important barrier for implementation. To formalize the role of the school district in the implementation process, a decision was made to add internal facilitators as an implementation strategy. Internal facilitators have been shown to support the implementation process, by among others overcoming obstacles for implementation [27, 28]. Core activities of implementation facilitators identified in the literature include for example problem identification, action/implementation planning, clarifying roles, goal/priority setting, and assessing, and monitoring implementation [29]. There is growing evidence for the effectiveness of implementation facilitation in improving implementation [30, 31] and facilitators have in several studies been shown to successfully facilitate implementation efforts [28, 32].

### The current study

A new cluster-randomized controlled trial will be conducted focussing on the processes through which the multifaceted implementation strategy is hypothesized to operate to affect the implementation outcome. Previous work suggested that the multifaceted strategy was no more successful than a single component strategy. The current study directly builds on this finding by focusing on mechanisms, the distinct processes that explain how and why an implementation strategy leads to implementation success. More specifically, the current study will rigorously test theoretically driven hypothesized mechanisms of change in the multifaceted strategy. This will significantly refine and expand previous findings because it allows for the examination of exactly how the multifaceted strategy can lead to improvements in implementation outcomes (or fail to do so). This quantitative assessment will be supplemented with qualitative work, which will provide additional practice-based insight for understanding how the hypothesized mechanisms explain effectiveness in this context, and what additional considerations may be important for understanding why one strategy outperforms another. Additional enhancements in the current cluster-randomized controlled trial include refinement of the psychometric properties of the implementation outcome measure, adding an additional component (internal facilitators) to the multifaceted strategy, and using a larger sample.

### Study aim

The aim of the study is to explore the implementation mechanisms through which a single-implementation strategy and a multifaceted implementation strategy operate to affect the primary implementation outcome, which is fidelity to the *Guideline For The Prevention of Mental Ill-Health At The Workplace.* The implementation mechanisms will be examined by exploring different causal pathways in line with the COM-B model and applying a mixed method design. The mixed method design will entail a qualitative study of implementation process embedded within the cluster-randomized controlled trail examining implementation mechanisms. The methods will be used in a complementary manner to get a full understanding of the implementation mechanisms. Through its exploratory nature, the study will provide valuable knowledge on how implementation strategies work (or fail) to affect implementation outcomes.

### Research questions

Q1. How do the implementation strategies affect capability, opportunity, and motivation over time?

Q2. Is the effect of the implementation strategies on fidelity to the guideline mediated by capability, opportunity, and motivation?

Q3. Does baseline readiness to change moderate the implementation strategies’ implementation mechanisms?

### Trial design

**Fig. 1:**
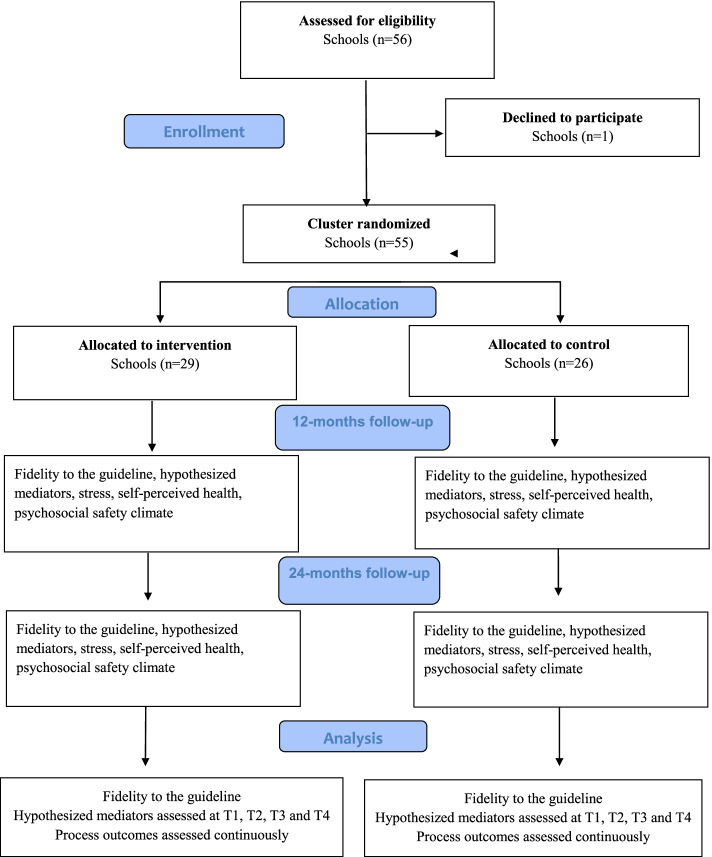
CONSORT flow-chart

The study has a cluster-randomized-controlled trial design with before and after measurements. Schools are randomized (1:1 ratio) to ARM 1 or ARM 2. ARM 1 receives all strategies during year 1, while ARM 2 forms implementation teams and receives the educational strategy in year 1 and the other strategies during year 2 (Fig. 1). The study is funded by the Swedish Research Council for Health, Working Life and Welfare, approved by the Swedish Ethical Review Agency (2021-01828) and registered at ClinicalTrials.org (dr.nr 2020-01214).

The description of the design applied in the protocol follows the CONSORT—and TIDier—reporting guidelines (see checklist in Additional files 1 and 2).

## Methods

### Study setting and population

The study is conducted among public primary and upper-secondary schools (*n*=55 schools) in four municipalities in Sweden. Two municipalities are located in an urban area and two municipalities in a rural area. The municipalities give a good representation of different geographical areas and socioeconomic as well as urban and rural areas.

### Eligibility criteria

All personnel employed by the schools are eligible to participate, including teachers, administrators, and support personnel (e.g., reading specialist, teacher’s aide, and paraprofessional). Individuals not employed by the school (e.g., external cleaning and maintenance personnel) will be excluded, as they do not fall under the management of the school.

### Interventions

#### Guideline to be implemented

The object that will be implemented is the *Guideline For The Prevention Of Mental Ill-Health At The Workplace* [5]. The guideline includes recommendations how employers in cooperation with their personnel can prevent mental ill health within their organization. The guideline includes the following recommendations: (1) workplaces have well-established routines/policies related to organizational and social risk management, (2) employers have knowledge of the relationship between organizational and social risks and mental ill health, and (3) workplaces regularly assess their organizational and social work environment and intervene on identified risk factors. Personnel involvement is strongly emphasised in the guideline. For example, it is recommended to conduct group discussions with personnel to prioritize work environment risks that need changing to prevent mental ill health and to involve personnel in the development of action plans describing changes that need to be made. The guideline was systematically developed in a collaboration between researchers, employer representatives, and occupational health service staff and includes recommendations that are based on the best available evidence in the field (e.g., [33–36]. The guideline complies fully with the Swedish Work Environment Authority’s organizational and social work environment (AFS 2015:4) provisions. Since 2018, the guideline is disseminated through the Swedish Agency for Work Environment and Expertise (https://sawee.se/). A full description of the recommendations has been published previously [9].

#### Implementation strategies

**Fig. 2:**
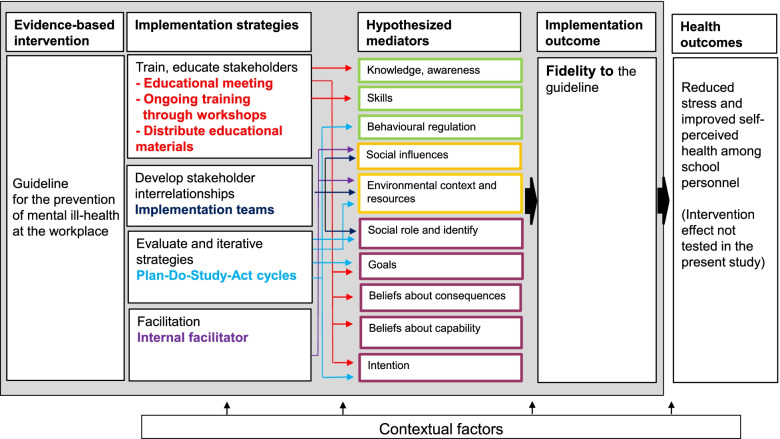
Implementation logic model

The strategies to be evaluated are based on those developed in our previous study [9]. An additional strategy was added, namely an internal facilitator. Refinements were made to the content of the educational meeting and workshops, with more focus on knowledge provision regarding the guideline recommendations and Plan-Do-Study-Act methodology. Moreover, educational material to support the formation of Plan-Do-Study-Act cycles was added in the form of templates to be used by the implementation team. To provide a deeper understanding of the mechanism of change, the original COM-B pathways were reassessed and refined based on the Theoretical Domains Framework (TDF) [37]. The domains of the TDF have previously been successfully mapped onto the COM-B, with excellent agreement [37]. Implementation strategies specified in accordance with Proctor recommendations for specification [38] are described in Table 1. The implementation logic model is depicted in Fig. 2.

**Table 1:** Specification of the implementation strategies

Implementation strategy	Actor(s)	Action	Action target	Dose	Timing
Organize implementation teams (48)—ERIC cluster Develop stakeholder inter-relationships	Headmaster	Forms an implementation team responsible for implementing the guideline within the school. Plan implementation team meetings to discuss guideline implementation	Team members (school principal and 3–4 representatives of school personnel, e.g., teacher union representative, health, and safety officer). - Create a structure for the implementation of the guideline, including the role clarity - Create an environmental context and allocate resources for implementation - Increase the social support between team members for implementing the guideline within the school.	Ongoing	Before the educational meeting
Conduct an educational meeting (15)—ERIC cluster Train and educate stakeholders.	Research team	Educate on mental ill health, the guideline, and the advantages of adhering to the guideline for the prevention of mental ill health. Facilitate goal formulation through group exercises aimed at formulating SMART goals.	Implementation team and school-district representatives - Increase the knowledge of the guideline - Form positive beliefs about the consequences of adhering to the guideline - Form positive beliefs about the capability to work in accordance with the guideline - Intent to implement the guideline - Form a goal related to implementing the guideline within their school	Once	Before the implementation of the guideline
Conduct ongoing training in the form of workshops (19)—ERIC cluster train and educate stakeholders	Research team	Provide training in the guideline and improvement cycles by including a mixture of lectures, discussions, and exercises. Facilitate knowledge sharing between the teams through workshop activities.	Implementation team, school-district representatives - Increase knowledge of the guideline and PDSA - Increase skills how to implement the guideline by using PDSA - Form positive beliefs about consequences of adhering to the guideline - Form positive beliefs about capability to implement the guideline - Intent to implement the guideline	5 workshops	First workshop will be held two weeks after the educational meeting
Conduct cyclical small tests of change (14)—ERIC cluster use evaluative and iterative strategies	Implementation team	Conduct Plan-Do-Study-Act cycles. Specify a Plan during workshops, identify barriers and facilitators, carry out the Plan between workshops (Do), analyze whether it went well and what needs to be improved (study), change the plan if needed (act) and plan the next cycle.	Implementation team - Set a goal for implementing the guideline - Plan for action by specifying how, where and by whom the guideline will be implemented - Increase the role clarity regarding implementation tasks. - Create an environmental context and allocate resources for implementation. - Create habitual behavior regarding implementation	Ongoing	The first Plan is developed during the first workshop
Distribute educational materials (21)—ERIC cluster train and educate stakeholders	Research team	Distribute educational materials during the meeting and workshops, including the guideline and accompanying material, slides of the presentations, working material that can be used to develop goals for implementation and PDSA templates.	Implementation team, school-district representatives - Increase the knowledge of the guideline - Increase the beliefs about capabilities to implement the guideline. - Facilitate goal setting and action planning	Six times	During the educational meeting and each workshop
Implementation facilitation (10)—ERIC cluster provide interactive assistance	School district representatives	Support the implementation teams during the educational meeting and workshops. Schedule meetings based on the needs of each implementation team.	The school district representative acts as an internal facilitator to the implementation teams. - Create an environment context and allocate resources within which knowledge is exchanged, barriers to implementation identified, and processes or solutions to overcome those barriers developed, applied, and refined. - Provide a social support to the implementation teams	Ongoing	Starts at the educational meeting

##### Implementation teams

At each school the headmaster will form an implementation team consisting of 4–5 people depending on the size of the school. The team will include the principal, teacher (union)—representative, occupational health and safety officer, and support staff representative. The members of the team should represent the actual mix of staff who will be involved in the implementation of the guideline. To support the principal with the formation of the team, instructions will be sent by email, including a template to specify team members’ names, roles, and motivation for inclusion. The team will be encouraged to meet regularly. The schools will choose the length and frequency of the meetings.

##### Educational meeting

At each municipality, implementation teams and school-district representatives (e.g., director of education, HR-specialist) will participate in a 1-day educational meeting conducted by an implementation researcher (LK) and a researcher in occupational health (CB). The educational meeting will be a mixture of lectures, discussions, activities, and group exercises. During the first lecture, the researchers will provide information on mental ill health, the guideline, and how working in accordance with the guideline can prevent mental ill health. Each team will conduct exercises aimed at reflecting on their current adherence to the guideline recommendations, identifying the benefits of adhering to the guideline, setting an implementation goal, and planning for implementation. Instructions related to the exercises will be given through several short lectures given by the implementation researcher and complementary materials, including templates for goal formulation. At the end of the meeting, a lecture will be given on what is needed to succeed with the developed plan. Potential barriers and facilitators are also introduced.

##### Ongoing training in the form of workshops

To support the implementation teams with their implementation process, five 2.5-h workshops will be held at each municipality by the researchers (LK and CB) over a 12-month period. Each workshop contains a lecture aimed at providing detailed information on guideline recommendations (one recommendation per workshop). The workshops will mix lectures with discussions, activities, and exercises. Each workshop is divided into three modules. During the first module, the teams will present the progress they have made with their PDSA-cycle and adjustments that need to be made to continue (see description below). The second module is aimed at providing detailed information on the guideline recommendations. This module includes lectures combined with discussions and exercises. During the exercises, teams compare how they currently work with the prevention of mental ill health with what is recommended by the guideline. At the end of the module, teams will have identified what needs to be changed. The third module is aimed at providing teams with information and skills regarding how to implement the guideline within their workplace. Lectures will be given on Specific, Measurable, Achievable, Realistic, and Timely (SMART)—goals and how to conduct PDSA cycles. During this module, teams will conduct exercises aimed at formulating goals and making plans for implementation in accordance with the PDSA cycles. During each workshop, implementation teams receive complementary materials, including handouts of the lectures, material, and templates for the exercises.

##### Plan-Do-Study-Act cycles

The implementation teams will start with their first PDSA cycle during the first workshop. In line with the PDSA structure, the teams will compare their current situation with the guideline recommendations, formulate a goal based on the recommendations, identify barriers to achieving the goal, identify what changes are needed to achieve the goal, and develop an implementation plan that describes how the changes are to be implemented and who is responsible for the changes. Between workshops, the teams are encouraged to implement the changes and measure the effects, compare the results with the formulated goal, and, if necessary, adjust the changes. To support the teams with their PDSA cycles, lectures will be given during the workshops explaining how to conduct PDSA cycles; moreover, teams will during each workshop receive PDSA planning templates. The template is based on the planning tool developed by the National Implementation Research Network and translated to Swedish for the purpose of the study [26]. At the end of each workshop, the teams will hand in a copy of their template to the research team. Between workshops, teams will be reminded to complete their template encouraging them to reflect over their implementation progress.

##### Internal facilitator

At each municipality a representative of the school district will be selected in collaboration with the research team to act as internal facilitator. The internal facilitator should be familiar with the school-level organizational structures and procedures. In this study, the functions of the facilitator can include supporting implementation teams with identifying changes to be made, helping to prioritize, supporting with identification, and understanding barriers, helping with problem-solving if needed, and providing positive reinforcement. Overall, the facilitator will provide support needed for to the implementation teams to work according to the recommendations of the guideline, for example, by providing resources and technical support. The internal facilitator will participate during the educational meeting and each workshop.

### Outcomes

Table 2 describes the measurement variables, method of data collection, data source, and time-point for each measure.

**Table 2:** Measurement variables, method of data collection, data source, and time points

Measure	Method of data collection	Data source	Time-point
**Implementation outcome**			
Fidelity to the guideline	Electronic checklist	School management	Baseline, 12- and 24-month follow-ups
	Web survey based on previous study [9]	School personnel	Baseline, 12- and 24-month follow-ups
Demographics: gender, age, education, occupation, work experience, years at workplace, frequency of overtime	Web survey	School management, school personnel	Baseline, 12- and 24-month follow-ups
**Hypothesized mediators**			
Knowledge	Web survey including DIBQ items [39]	Participants exposed to the implementation strategies	During the educational meeting, directly after workshops 2 and 5, and at a 12-month follow-up.
Skills	Web survey including DIBQ items [39]	Participants exposed to the implementation strategies	During the educational meeting, directly after workshops 2 and 5, and at a 12-month follow-up
Social/professional role and identity	Web survey including DIBQ items [39]	Participants exposed to the implementation strategies	During the educational meeting, directly after workshops 2 and 5, and at a 12-month follow-up
Beliefs about capabilities	Web survey including DIBQ items [39]	Participants exposed to the implementation strategies	During the educational meeting, directly after workshops 2 and 5, and at a 12-month follow-up
Beliefs about consequences	Web survey including DIBQ items [39]	Participants exposed to the implementation strategies	During the educational meeting, directly after workshops 2 and 5, and at a 12-month follow-up
Intentions	Web survey including DIBQ items [39]	Participants exposed to the implementation strategies	During the educational meeting, directly after workshops 2 and 5, and at a 12-month follow-up
Goals	Web survey including DIBQ items [39]	Participants exposed to the implementation strategies	During the educational meeting, directly after workshops 2 and 5, and at a 12-month follow-up
Environmental context and resources	Web survey including DIBQ items [39]	Participants exposed to the implementation strategies	Directly after workshops 2 and 5, and at a 12-month follow-up
Social influences	Web-survey including DIBQ items [39]	Participants exposed to the implementation strategies	Directly after workshops 2 and 5, and at a 12-month follow-up
Behavioral regulation	Web survey including DIBQ items [39]	Participants exposed to the implementation strategies	Directly after workshops 2 and 5, and at a 12-month follow-up
**Moderator**			
Readiness to implement	Leader Readiness to Implement Tool (LRIT) items (Cook et al., submitted)	School management	Before the educational meeting, during workshop 5
	Staff Readiness to Implement Tool (LRIT) items (Cook et al., submitted)	Implementation team members	Before the educational meeting, during workshop 5
**Process outcomes**			
Penetration	Participation list	Research team	During the educational meeting and workshops
Fidelity	Observation, meeting notes, work documents	Research team	Continuously
Implementation process	Semi-structured interviews	Principals and members of the implementation team	12- and 24-month follow-ups
Context	Follow-up phone call	Principals	6 months after the educational meeting
**Descriptive variables**			
Demographics: gender, age, education, occupation, work-experience, years at workplace, frequency of overtime	Web survey	School management, school personnel	Baseline, 12- and 24-month follow-ups
Health outcomes: general health and self-perceived stress	Web survey [40–42]	School management, school personnel	Baseline, 12- and 24-month follow-ups
Psychosocial safety climate	Web survey [43]	School management, school personnel	Baseline, 12- and 24-month follow-ups

### Participant timeline

**Fig. 3:**
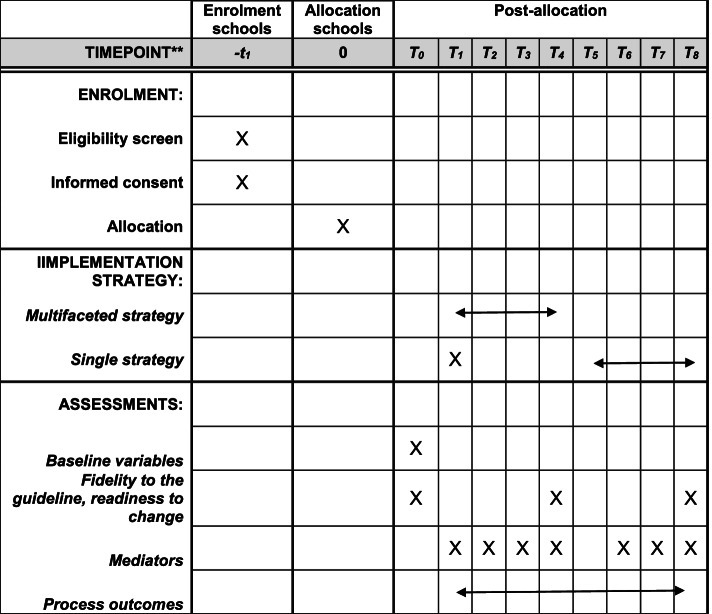
The time schedule of enrolment, implementation strategies, and assessments

The time schedule of enrolment, implementation strategies, and assessments is described in Fig. 3.

### Sample size and power

For the mediation analysis, the power calculation by Lee et al. [44] is used due to the lack of information on the mediators to conduct own calculation. In the study by Lee and colleagues, the same TDF constructs as well as a similar modeling strategy were used to assess mediators as in the present study. Their calculation indicated that a sample of 121 provides 80% power to detect moderate treatment mediator and mediator outcome effects. With 55 participating schools and 4 to 5 implementation team members per school, we are expecting a sample size of around 220 participants, clustered in their respective schools, for mediation analysis. However, for the implementation outcome, we will have data for all school personnel, with an estimated sample size of around 1500 participants (equivalent to 28 participants per school).

### Recruitment

Recruitment of municipalities started in September 2020 by contacting municipalities that had previously shown interest in participating in research projects, advertising the project via stakeholder-related channels, and contacting municipalities by email. Between September 2020 and April 2021, the research team met with four municipalities that showed interest in participation. All municipalities agreed to participate. Next, informational meetings were held with principals and union representatives to inform them about the background, rationale, and logistics of the project. Finally, school personnel were recruited. An informational film recorded by the research team describing the project and what participation entails was disseminated to all personnel by the principals. An informational letter describing the study objectives, research approach, voluntary participation, data collection process, and that participation can be stopped at any time on request by the participants was sent by email. School personnel were given the opportunity to contact the research team if anything was unclear. Individuals who decided to participate in the study accordingly completed an informed consent form.

### Assignment of interventions

Due to practical necessity (spread of recruitment), schools were randomized in two rounds. The first randomization was stratified by municipality and clustered, so that all schools in a school district with same central leadership or alternatively all schools with the same headmaster were randomized as a cluster. Upper secondary schools were randomized as a separate stratum in the first randomization round to guarantee their equal distribution among the two groups. The second round of randomisation included only one municipality. Two school pairs (four schools in total) had the same principles and were randomized as clusters. These schools were randomized as a separate stratum from the other schools to maintain balance in the number of pupils and staff. No further stratifications were made. The randomization was done by randomly ordering the clusters within the strata and assigning group allocation based on order. A seed was set for replication. Randomization was conducted by an independent statistician, blind to the identity of the schools and not involved within the project. The principal investigator was not involved in the group assignment. Due to logistical reasons (i.e., planning workshops within the school’s schedules) randomization occurred prior to baseline measurements. The principal investigator informed the municipalities and schools of their group allocation. Blinding of principals or school personnel is not possible within the chosen study design.

### Data collection methods

#### Fidelity to the guideline

Fidelity to the guideline will be assessed by a checklist and web survey at baseline and 12- and 24-month follow-ups. The checklist contains three sections and 12 statements with one section for each guideline recommendation. Per recommendation, the school management indicates their agreement with the related statement, e.g., “At our school, we have updated work environment policies”. Respondents indicate whether they agree with the statement and if so, provide a detailed description of how and when the activity was performed, and attach the related documents (e.g., work environment policies). The checklist is electronic, developed for the purpose of the study, and was pilot-tested among a small sample of principals not participating in the study.

The web survey provides a measure of fidelity to the guideline from the perception of the school personnel. It is hypothesized that if schools adhere to the guideline recommendations, then the school personnel will be exposed to the related activities. The web survey was developed for our previous study [9]. For the present study, cognitive testing of the questions and response categories was conducted among teachers (*n*=5), to ensure that the questions successfully capture the scientific intent and that they make sense to the respondents. The survey contains 12 statements related to the recommendations of the guideline. Respondents indicate on a 5-point Likert Scale ((1) “strongly disagree”- (5) “strongly agree”) the extent of their agreement with the statements. For example, “During the last work environment survey results of the survey were presented to personnel (e.g., by email or during a joint meeting)”. A link to the web survey will be sent by e-mail by the project team. The survey can be obtained upon request from the corresponding author.

#### Hypothesized mediators

Mediators will be assessed with the Determinants of Implementation Behaviour Questionnaire based on the Theoretical Domains Framework (DIBQ [39]). For this study, items were translated and back-translated from English to Swedish by an independent researcher. The items were pilot-tested among participants of our previous school study. The questionnaire will be completed on four occasions by all participants exposed to the implementation strategies. The following domains will be assessed, each with three items, on a 7-point scale ranging from (1) strongly disagree to (7) strongly agree: knowledge, skills, beliefs about consequences, beliefs about capability, social influences, intention, goals, behavioural regulation, and environmental context and resources. Table 2 provides an overview of the time points when each specific domain will be assessed.

#### Descriptive variables

Descriptive variables, including age, gender, number of years working at the school, and number of years working within the profession, will be assessed by web survey completed by principals and school personnel at baseline and 12- and 24-month follow-ups. Self-reported stress and self-rated health will be assessed with a single item with 5-point response anchors ranging from (1) “not at all” (5) “very much” [40, 41]. Self-perceived health is assessed with a single question from the SF-12 Health Survey [42] with 5-point response anchors ranging from (1) excellent to (5) bad. Self-reported psychosocial safety climate (PSC) will be assessed with the 4-item Psychosocial Safety Climate Scale with 5-point response anchors ranging from (1) strongly disagree to (5) strongly agree [43].

#### Readiness to implement

Readiness to implement will be assessed with the Leader/Staff Readiness to Implement Tool during the educational meeting and during workshop 5. The tool was developed for use in the school context and has a leader and staff version (Cook et al., submitted). Both versions contain 14 items with 5-point response anchors ranging from (1) strongly disagree to (5) strongly agree.

#### Process outcomes

The implementation process will be evaluated by collecting information on implementation outcomes as defined by Proctor and colleagues [45]. Implementation outcomes include penetration and fidelity. Moreover, we will explore participants’ perspectives regarding how or why the implementation strategies worked or failed (and how they might be optimized in subsequent efforts) via observations and semi-structured interviews.

##### Penetration

Penetration will be assessed through attendance lists, completed by the research team during the educational meetings and during each workshop. Penetration will be operationalized as the absolute number and proportion of individuals participating in the educational meeting and workshops in relation to those expected to participate. Information on penetration is collected as a low penetration could influence the functioning of the implementation mechanisms.

##### Fidelity

Fidelity to the educational meeting and workshops will be assessed by the research team during the meeting and workshops by using a checklist to note whether they were implemented in accordance with the study protocol. A research log is kept describing deviations and possible reasons for deviations. Fidelity to the implementation teams will be assessed during the educational meeting by examining whether each school has formed an implementation team that is comprised of the recommended representatives. Throughout the study, information will be collected on whether the formation of the teams has changed along with possible reasons for those changes. Fidelity to the PDSA cycles will be assessed by collecting the implementation teams’ PDSA templates after each workshop and comparing the templates against the key principles of the strategy [46]. Fidelity to the internal facilitator will be assessed by checking whether each municipality has appointed an internal facilitator. Information on fidelity is collected as low fidelity to the different strategies could influence the functioning of the implementation mechanisms.

##### Implementation process

The functioning of the implementation strategies will be assessed by observation and by semi-structured interviews. Observations of the implementation teams will be made during workshops 2 and 5 by using an observation protocol (based on [47]) focusing on the following themes: planning and organisation, interest and engagement, productivity, process, and group-dynamic and climate. The collected information will be used to assess the functioning of the implementation teams. Semi-structured interviews will be conducted at a 12-month follow-up with all principals and a purposive selection of implementation team members based on their role in the implementation process. An interview guide will be developed for the purpose of the study covering the following themes: activities related to the implementation process, how the implementation strategies have supported the implementation process, challenges experienced during the implementation process, how implementation strategies might be optimized in future efforts, additional strategies that might be needed, and other areas for improvement. Semi-structured interviews will also be conducted with school district representatives to assess the extent to which they have acted as internal facilitators supporting the implementation process among their schools according to their functions. The interviews will be conducted by the research team, audio-recorded, and transcribed verbatim.

##### Context

Six months after the educational meeting the research team will conduct a telephone follow-up (20–30 min) with all principals to assess contextual factors potentially influencing the functioning of the implementation mechanisms. For this study, an interview guide was developed based on an existing guide [24], which was adapted to fit the school context. The guide includes two questions and follow-up prompts. First, the principals will be asked to summarize the activities that they have undertaken to implement the guideline since the educational meeting. Second, they will be asked to identify any circumstances that may have occurred at their school and/or school district and may have influenced the implementation process, such as personnel turnover and organizational changes. The follow-ups will be audio-recorded and transcribed verbatim.

### Data management

Data will be collected by using the secure web platform Research Electronic Data Capture (RedCap [48]) and stored electronically in a password-protected folder on a secure server at our institute. All data will be deidentified, by removing all person-identifiable information from the database and replacing it with a code. The code key is saved, which will enable individuals to request an extract of the collected data and demand that information on him/her will be destroyed without any given reason. Only the research team will have access to the identification code, which is stored separately from the data.

### Data analysis

The study will use mixed methods to fulfil the complementarity function for evaluation purpose [49]. Thus, causal linkages between the strategies, mediators, and implementation outcomes will be tested with the help of quantitative methods, while data from semi-structured interviews will be analyzed to provide further insight into the process of the mechanism functioning and experiences of those involved. The results of the qualitative study of the implementation process will be nested within the quantitative study of implementation mechanisms.

### Statistical analysis

Categorical variables will be presented with count and percentage, continuous variables with median and interquartile range, unless the mean and standard deviation is deemed more informative. The primary outcomes of interest are the mediated effects between the outcome fidelity and the implementation strategy. The mediators are the domains of the Determinants of Implementation Behaviour Questionnaire (DIBQ) [39]. All the domains consist of three items. The first step will be to use a confirmatory factor analysis to confirm that the hypothesized domains are reasonable and can be used. We will then use a mediation analysis to estimate the average mediation effect, average direct effect, and average total effect as well as the proportion mediated. The mediation analysis will be done within the Structural Equations Modeling framework to best take advantage of the data structure. A detailed statistical analysis plan will be developed in collaboration with a statistician prior to starting the data analysis.

### Ethics

The study has been approved by the Swedish Ethical Review Authority (No. 2021-01828). The study complies fully with current ethical requirements regarding the handling and storage of personal data and regarding the informed consent process in accordance with General Data Protection Regulation. An information letter is sent to potential participants describing the study aim, research approach, data collection process, and voluntary participation, including the possibility to withdraw at any time. The letter also states that data is only collected for the purpose of the study, presented at the group level and that no personal data will be shared with the school or school district. The letter allows participants to make informed decisions about whether to participate. Informed consent will accordingly be collected from all participants.

### Plans for dissemination

Results will be disseminated within the scientific community through peer-reviewed open-access papers and scientific conferences. Results will also be disseminated outside of the research community, for example, through social media, popular scientific reports, and national seminars for key stakeholders, including municipalities and schools.

## Discussion

The present study will further our understanding of how implementation strategies work (or fail) to affect implementation outcomes. This study will provide both knowledge on the impact of implementation strategies on theorized mediators and on whether the impact on these mediators facilitates implementation. Ultimately, the knowledge gained will aid the selection of effective implementation strategies that fit specific determinants, which is a priority for the field [50]. Despite recent initiatives to advance the understanding of implementation mechanisms [11], studies testing these mechanisms are still uncommon [11–13, 51].

The study has several strengths. First, to assess implementation mechanisms, we will conduct a cluster-randomized controlled trial exploring different causal pathways of how a multifaceted implementation strategy compared to a discrete strategy impacts the fidelity to the guideline via hypothesized mediators originating from the COM-B model. The need for randomized controlled trials with high-quality designs testing implementation strategies and articulating and evaluating theory-derived mechanisms has been underscored in several reviews [12, 13]. Second, implementation mechanisms will be explored with mixed methods. Few studies have used a mixed methods approach to understanding implementation mechanisms [13]. Finally, this study will provide valuable knowledge on how implementation strategies work in a school context. Most implementation effectiveness studies are conducted within a health care setting, emphasizing the need for school context-specific studies [52]. As the Swedish school setting shares many similar features with other countries’ school contexts, we believe that the knowledge gained in the present study will be generalizable to school systems outside of Sweden.

## Supplementary Information

**Additional file 1.** The TIDieR (Template for Intervention Description and Replication) Checklist.

**Additional file 2: Table 1**. CONSORT 2010 checklist of information to include when reporting a cluster randomised trial. **Table 2**. Extension of CONSORT for abstracts to reports of cluster randomised trials.

## Data Availability

Not applicable
